# No evidence of jugular venous valve insufficiency in patients with migraine – a controlled study

**DOI:** 10.1186/1129-2377-14-52

**Published:** 2013-06-19

**Authors:** Kasja Rabe, Michael Küper, Dagny Holle, Irini Savidou, Oliver Kastrup, Andreas Jähne-Blasberg, Hans Christoph Diener, Zaza Katsarava, Markus Frings

**Affiliations:** 1 Department of Neurology and Headache Center, University of Duisburg-Essen, Hufelandstr, Essen, 55, 45122, Germany; 2 Department of Neurology, Evangelisches Krankenhaus Unna, Massener Straße 84, Unna, 59423, Germany

**Keywords:** Migraine, Transient global amnesia, Jugular venous valve insufficiency, Ultrasound

## Abstract

**Background:**

Migraine with aura is associated with patent foramen ovale and right-left-shunt. Jugular venous valve insufficiency is a further vascular anomaly. It is a frequent finding in transient global amnesia which is associated with migraine. Therefore, we investigated the prevalence of jugular venous valve insufficiency in migraine.

**Methods:**

Subjects included in this study were participants of the population based German Headache Study on the prevalence of primary headaches. In 36 patients with migraine with aura, 50 patients with migraine without aura and 43 controls without headaches duration of backward venous flow, peak velocity flow and diameters of the jugular venous valves were assessed by color-coded duplex and Doppler sonography and compared between groups. In all migraine patients, examination was performed between and not during migraine attacks. Therefore, 9 additional patients with chronic daily headache were investigated during headache.

**Results:**

We did not find statistically significant differences in duration of flow, peak velocity flow and diameter of the jugular venous valves in patients with migraine with aura (mean values 0.53 ± 0.43 sec; 35.47 ± 33.87 cm/sec; 8.84 ± 3.17 mm), migraine without aura (0.61 ± 0.63 sec; 33.39 ± 25.80 cm/sec; 8.15 ± 3.02 mm) or controls (0.64 ± 0.51 sec; 35.28 ± 31.76 cm/sec; 8.79 ± 2.97 mm) (group effects p-values >0.41). For all parameters results were the same for the left and the right side of jugular venae (side effects p-values >0.09). Also patients with chronic daily migraine with headache during the examination showed no differences to controls (0.52 ± 0.49 sec; 27.95 ± 21,75 cm/sec; 8.07 ± 2.71 mm) (all p-values > 0.23).

**Conclusions:**

The prevalence of internal jugular venous valve insufficiency is not increased in persons with migraine.

## Background

Migraine is associated with a number of vascular conditions such as stroke or myocardial infarction [1]. There seems to be an association with migraine with aura and right – left shunts and patent foramen ovale (PFO) [2]. Some authors have suggested that a migraine aura could be caused by desoxygenated blood that reaches cerebral circulation by a right-left shunt [3]. However, whether this association is causal or coincidental or if migraine and PFO share a common genetic predisposition is not known.

Internal jugular venous valve insufficiency (JVVI) is another vascular anomaly that is a frequent finding in transient global amnesia (TGA) [4-11], a disorder with a high prevalence of associated migraine [12]. TGA is characterized by an inability to form new memories, with an acute onset of anterograde and retrograde amnesia that lasts 1 to 24 hours [5-12]. The etiology and pathophysiology of TGA are still not completely understood. JVVI could cause impaired venous drainage of intracranial veins through increased thoracic pressure that could lead to a hippocampal venous congestion [5]. This is supported by the observation that TGA often follows Valsalva-maneuvers or physical activity in untrained subjects.

TGA and migraine on the one hand and TGA and JVVI on the other hand are independently associated with one another. Aim of this controlled trial was to investigate whether migraine with or without aura is associated with JVVI using color-coded duplex and Doppler sonography.

## Methods

### Data collection and questionnaire

**Figure 1:**
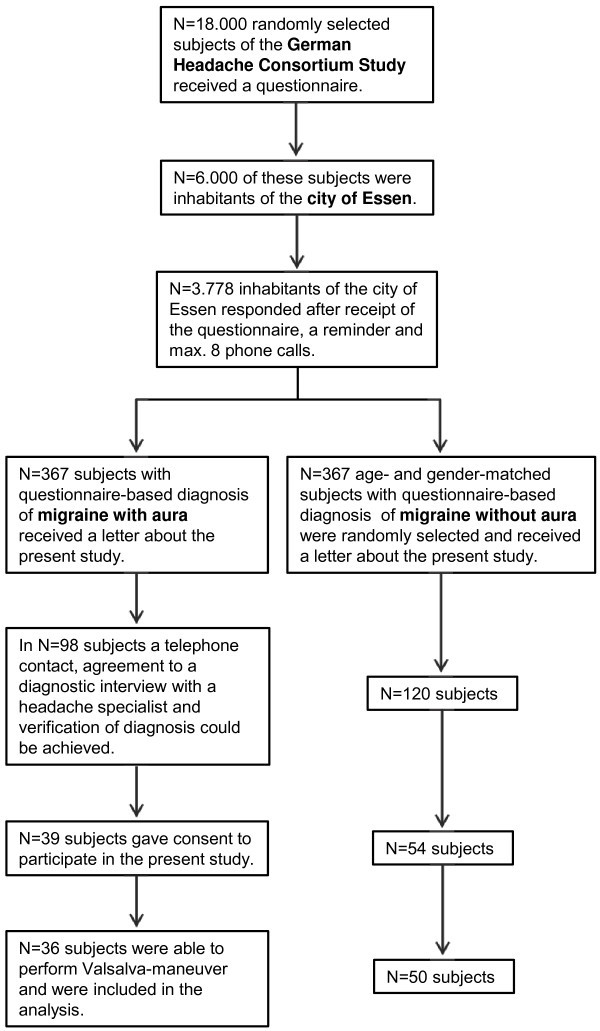
Flowchart of the procedure of subject recruitment for subjects with migraine with und without aura.

Patients included in this study were recruited from the population based German Headache Study on the prevalence of primary headaches. Baseline data of a population of randomly selected 18.000 subjects were assessed between 2003 and 2005 [13]. The study questionnaire used to classify headache was constructed based on the criteria of the International Headache Society and prospectively validated [14]. The specificity and sensitivity for migraine diagnosis for this questionnaire was 0.85 in relation to a diagnosis based on an examination by a headache specialist [15]. Subjects of the population of the German Headache Study living in Essen diagnosed with migraine with and without aura and willing to take part in further studies were invited to this sub-study in the years 2008 to 2010. Some of the patients also participated in an earlier study of our group [16]. Patients were invited for an interview during which the diagnosis of migraine and aura status were verified according to the IHS-criteria by one of three headache specialists (MK, DH, KR). Figure 1 provides more details about the number of subjects at each stage of the study. Controls were recruited from the hospital staff and acquaintances of the study group without headaches. To ensure that controls did not suffer from relevant headache, medical history including history of potential headaches was assessed by the same three headache specialists (MK, DH, KR). The local ethics committee approved the study. All participants gave written informed consent prior to study inclusion. The study was performed in accordance to the Declaration of Helsinki. The institutional review board that approved the study is: Ethic committee of the University Duisburg Essen.

### Subjects

Out of the included 138 subjects 129 persons were eligible for statistical analyses. Nine subjects were not able to perform the Valsalva-maneuver sufficiently. 36 persons had migraine with occasional aura symptoms (28 females, 8 males; mean age 50.5 yrs. (27 to 70 yrs.), 50 persons had migraine without aura (36 females, 14 males; mean age 50.3 yrs. (25 to 68 yrs.) and 43 persons did not have any headaches (35 females, 8 males; mean age 51.8 yrs. (28 to 68 yrs.)). Headache frequency in subjects with migraine with aura was 3.4 ± 3.4 days/month (range 0–12) and in subjects with migraine without aura 3.3 ± 3.0 days/month (range 0–10). Headache frequency did not differ between subjects with migraine with or without aura (F(1,84) = 0.59, p = 0.45; one-way ANOVA). Among the subjects with migraine with aura n = 29 had visual, n = 12 sensory, n = 4 aphasic, n = 2 motor and n = 1 auditory aura. In 12 subjects, two different types of aura were present. Gender and age between subjects with migraine with and without aura and controls was not significantly different (gender: F(2,126) = 0.58, p = 0.56; age: F(2,126) = 0.26, p = 0.77; one-way ANOVA).

### Venous ultrasound examination

**Figure 2:**
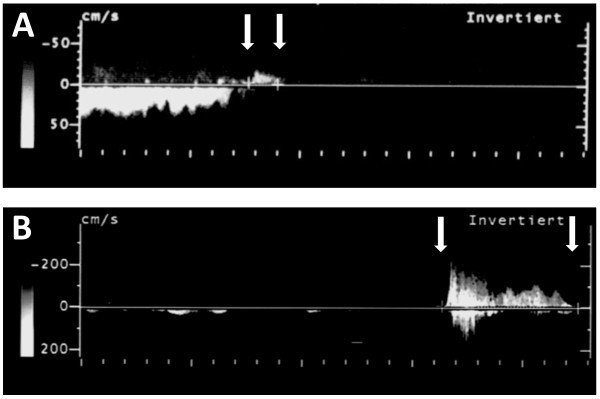
**Doppler sonography of venous valve flow during closure. A** shows Doppler sonography of venous flow during closure of a competent jugular venous valve by Valsalva maneuver. The arrows indicate the beginning and the end of the backward flow. After valve closure no orthograde venous flow appears because of an ongoing Valsalva maneuver over several seconds. **B** shows prolonged backward flow in an insufficient valve. Notice the different graduation of the scale of flow velocity revealing a higher peak velocity flow in the insufficient valve.

Jugular venous valve closure was assessed by color-coded duplex sonography (Acuson Antares, Siemens AG, Erlangen, Germany; 4–9 MHz linear array transducer). Subjects were asked to perform a pressure-controlled Valsalva-maneuver that induced closure of the jugular venous valves. In competent valves without any signs of insufficiency the venous reflux is very short. A short backward flow is observed before cessation of blood flow Figure 2A. In insufficient valves a prolonged backward flow is seen Figure 2B [17]. Duration of backward flow, peak velocity flow and diameters of the jugular venous valves were assessed and compared between groups. Valve performance was evaluated using a cut-off value according to a study by Nedelmann et al. [17]. In this study, 1.23 sec were the shortest duration of retrograde flow in insufficient valves. Valves with a backward flow of more than 1.23 sec were considered to be insufficient. In another study by Nedelmann et al. [9] the cut-off value for insufficient valves was set to 0.88 sec according to a three time standard deviation of duration of backward flow in competent valves. In the present study, the number of insufficient valves was determined for this second, less conservative cut-off value, too.

### Statistical analysis

Statistical analysis of the data was performed using SPSS 20.0.0 (SPSS Inc., Chicago, III., USA). Three multifactorial analyses of variance (ANOVA) were calculated using duration of flow, peak velocity flow or diameter of valve as the dependent variable. For each ANOVA the side of the jugular venae (left and right) was within-subject factor and group (no headache, migraine with aura, migraine without aura) was between-subject factor. P values < 0.05 were considered significant. Bonferroni adjustments were applied when necessary with the significance level set to p < 0.016 (0.05/3 dependent variables). Data were normally distributed (Kolmogorov-Smirnov test).

## Results

In 18 subjects jugular valves were only detected on the left or right, but not on both sides (in 6 subjects with migraine with aura, in 5 subjects with migraine without aura, in 7 control subjects only on one side; in 10 subjects only on the left, in 8 subjects only on the right side). Therefore, jugular valves from 30 subjects with migraine with aura, 45 subjects with migraine without aura and 36 control subjects could be included in the statistical analysis.

A multifactorial analysis of variance did not show significant differences in duration of flow between subjects with migraine with or without aura and controls (group effect F(2,108) = 0.85, p = 0.43). Comparing the left and right side no side effect (F(1,108) = 2.86, p = 0.09) and no group by side effect (F(2,108) = 0.47, p = 0.62) was shown. Correspondingly, no differences in velocity of flow were found between groups (F(2,108) = 2.30, p = 0.79), between left and right side (F(1,108) = 0.001, p = 0.98) and comparing left and right side between groups (group by side effect F(2,108) = 1.38, p = 0.26). Finally, we could not find any difference in the diameter of the jugular venous valve between groups (F(2,108) = 0.89, p = 0.41). Moreover, comparing the left and right side no side effect (F(1,108) = 0.35, p = 0.56) and no group by side effect (F(2,108) = 2.44, p = 0.09) was shown (Table 1).

**Table 1:** Mean values and standard deviations (STD) for duration of flow, peak velocity flow and diameter of the jugular venous valve (JVV) for patients with migraine with aura (MA), migraine without aura (MO) and controls

	**Side**	**MA**		**MO**		**Controls**	
		**Mean**	**STD**	**Mean**	**STD**	**Mean**	**STD**
Duration of flow [sec]	Left	0.57	0.47	0.69	0.78	0.66	0.54
	Right	0.48	0.38	0.52	0.41	0.61	0.47
	Both	0.53	0.43	0.61	0.63	0.64	0.51
Peak velocity flow [cm/sec]	Left	32.71	29.98	37.67	26.66	33.39	26.67
	Right	38.49	37.93	29.27	24.52	37.32	36.74
	Both	35.47	33.87	33.39	25.80	35.28	31.76
Diameter of JVV [mm]	Left	8.94	2.97	7.57	3.05	8.95	3.11
	Right	8.72	3.43	8.72	2.91	8.64	2.87
	Both	8.84	3.17	8.15	3.02	8.79	2.97

Data are presented separately for the left and right side and as a mean value of both sides.

The number of insufficient jugular venous valves according to a cut-off parameter of 1.23 sec [17] was determined. Insufficiency was not found more frequently in any of the groups. 8% of subjects with migraine with aura symptoms, 11% of subjects with migraine without aura and 10% of subjects without headaches presented an either right, left or both sided insufficient jugular venous valve. Even according to a less conservative cut-off parameter of 0.88 sec [9], no differences between groups could be found. Considering this cut-off value, 14% of subjects with migraine with aura symptoms, 17% of subjects with migraine without aura symptoms and 18% of subjects without headaches presented insufficient jugular venous valves. Comparing the number of insufficient valves in all groups no significant differences could be found (p-values > 0.3, one-way ANOVA).

In all migraine patients, venous ultrasound examination was performed between and not during migraine attacks. To investigate the influence of this interictal approach, 9 additional patients with chronic daily migraine were examined during migraine headache (8 females, 1 male; mean age 40.4 yrs. (range 31 to 52 yrs.)). In these patients, duration of flow was 0.45 ± 0.25 sec on the left and 0.60 ± 0.69 sec on the right side (mean value of both sides 0.52 ± 0.49 sec), peak velocity flow was 26.18 ± 16.03 cm/sec on the left and 29.97 ± 28.19 cm/sec on the right side (mean value of both sides 27.95 ± 21.75 cm/sec) and diameter of the jugular venous valve was 6.96 ± 2.03 mm on the left and 9.17 ± 2.97 mm on the right side (mean value of both sides 8.07 ± 2.71 mm). A multifactorial ANOVA comparing these patients with the control group did not show significant group, side or group by side effects for all three dependent variables (all p-values > 0.23). Only one of these patients presented an insufficient jugular venous valve considering a cut-off parameter of 1.23 sec for duration of retrograde flow (11%) and two patients considering a cut-off parameter of 0.88 sec (22%).

No significant group differences were found in the present study. To assess the probability a type II error (accept null hypothesis H0 although an effect is actually present), i.e. the test power, post hoc power analysis was performed using Power software [18]. The test power to detect a difference between patients with migraine and controls in duration of backward flow, peak velocity flow and diameters of the jugular venous valves was determined. “Post-hoc” was entered as the type of power analysis and “other F-tests” as type of statistical test. Alpha error probability was chosen at 0.05, degree of freedom was determined at 2 and total sample size was 111. According to effect size conventions “effect size” was chosen 0.1 for small effects, 0.25 for medium effects and 0.4 for large effects. A test power of >0.8 was considered to be sufficient to accept H0. Our analysis of variance concerning the group effect revealed an adequate power to detect a large effect size (0.97), but not a medium (0.63) or small effect size (0.13).

## Discussion

This is the first study to assess the prevalence of JVVI in patients with migraine with and without aura. We could show that this vascular condition is not more prevalent in patients with migraine with aura or with migraine without aura than in control subjects without headache. Duration of retrograde flow, peak flow velocity and diameter of jugular venous valve that indicate JVVI were not significantly different between groups. Also patients with chronic daily migraine with headache during the examination showed no differences to controls. Taking different cut-off values for retrograde flow, JVVI was not significantly more frequent in one of the groups.

JVVI was assessed because it is frequently found in TGA [5,9,10] and migraine is a frequent comorbidity of patients with TGA (20-30%) [12,19]. TGA and migraine, and especially the confusional migraine [20] have clinical similarities. Headache (10-40%), nausea and dizziness are observed during a TGA. Both have a sudden onset and are completely reversible. They have a benign course and can be induced through external triggers [12,19,21]. However, the fact that migraine attacks preliminary occur in younger age groups and TGA in older age groups argues against a direct relationship. Another difference is the high frequency of migraine attacks during lifetime and the solitary event of TGA in most patients.

It is assumed that increased thoracic pressure during a Valsalva-maneuver leads to cerebral venous congestion due to a venous reflux by jugular venous valve insufficiency inducing focal hippocampal ischemia in TGA-patients. Just recently, the pathophysiology was questioned because intracranial venous flow seems to be unaffected [6]. Moreover, the cortical spreading depression“, a wave of cellular depolarization in the cerebral cortex that is discussed to mirror the aura in migraine, is proposed to be the pathophysiological equivalent of TGA [22-24].

If the incidence of JVVI would have been increased in patients with migraine, this would have argued against a causal relationship between migraine and PFO on the one hand and TGA and JVVI on the other hand. Detection of both pathologies in both disorders would have strengthened a coincidence without causality or a common genetic basis for these paroxysmal disorders and vascular anomalies. However, results of the present study did not show a higher prevalence of JVVI in migraine and previous studies did not show a higher prevalence of PFO in TGA. A first study suggested a relationship between TGA and PFO [25], but results could not be reproduced [26,27].

The strength of our study is the recruitment of patients from a population-based sample. The small sample size of the trial limits its validity. We cannot exclude that group-differences might be observed only in a larger group size. Additionally, differences in assessment of JVVI in sonography might have led to a bias. We defined JVVI according to a study of Nedelmann et al. [17] as duration of retrograde flow of at least 1.23 sec. Nedelmann visually differed between insufficient and sufficient valves and set criteria accordingly. He found insufficiency in 29% of healthy subjects, while we observed it only in 10%. Even if calculating less conservatively and taking a cut-off of 0.88 sec as was done in another study of Nedelmann in TGA-patients [9], only 18% of our healthy controls are characterized by insufficient valves. Sander et al. [10] found an even higher prevalence of JVVI in healthy subjects (40%). Differences between studies might be explained by examiner-related determination of duration of flow.

## Conclusion

In conclusion, the prevalence of JVVI is not increased in patients with migraine. We suggest that further clinical trials should be undertaken to address other vascular conditions in migraine.

## Abbreviations

PFO: patent foramen ovale; JVVI: jugular venous valve insufficiency; TGA: transient global amnesia.

## Competing interests

KR, MK, DH, IS, OK, AJB, ZK und MF have no competing conflict of interest. HCD has no competing interest, but received honoraria for participation in clinical trials, contribution to advisory boards or oral presentations from: Addex Pharma, Allergan, Almirall, Autonomic Technology, AstraZeneca, Bayer Vital, Berlin Chemie, Böhringer Ingelheim, Bristol-Myers Squibb, Coherex, CoLucid, GlaxoSmithKline, Grünenthal, Janssen-Cilag, Lilly, La Roche, 3 M Medica, Medtronic, Menerini, Minster, MSD, Neuroscore, Novartis, Johnson & Johnson, Pierre Fabre, Pfizer, Schaper and Brümmer, Sanofi, St. Jude and Weber & Weber. Financial support for research projects was provided by Allergan, Almirall, AstraZeneca, Bayer, GSK, Janssen-Cilag, MSD and Pfizer. Headache research at the Department of Neurology in Essen is supported by the German Research Council (DFG), the German Ministry of Education and Research (BMBF) and the European Union. HCD has no ownership interest and does not own stocks of any pharmaceutical company.

## Authors’ contributions

KR, MK, DH and IS have acquired the data. OK, HCD, ZK and MF have made substantial contributions to conception and design. KR, AJB and MF have made substantial contributions to analysis and interpretation of data. KR, MK, DH, IS, OK, AJB, HCD, ZK and MF have been involved in drafting the manuscript or revising it critically with important intellectual impact. All authors read and approved the final manuscript.
